# Effects of DARPP-32 Genetic Variation on Prefrontal Cortex Volume and Episodic Memory Performance

**DOI:** 10.3389/fnins.2017.00244

**Published:** 2017-05-11

**Authors:** Ninni Persson, Jonas Persson, Catharina Lavebratt, Håkan Fischer

**Affiliations:** ^1^Department of Psychology, Stockholm UniversityStockholm, Sweden; ^2^Aging Research Center, Karolinska Institutet and Stockholm UniversityStockholm, Sweden; ^3^Department of Molecular Medicine and Surgery, Karolinska Institutet and Center for Molecular Medicine, Karolinska University HospitalStockholm, Sweden

**Keywords:** DARPP-32, episodic memory, PPP1R1B (DARPP32), rs879606, rs907094, rs3764352, dopamine, glutamates

## Abstract

Despite evidence of a fundamental role of DARPP-32 in integrating dopamine and glutamate signaling, studies examining gene coding for DARPP-32 in relation to neural and behavioral correlates in humans are scarce. Post mortem findings suggest genotype specific expressions of DARPP-32 in the dorsal frontal lobes. Therefore, we investigated the effects of genomic variation in DARPP-32 coding on frontal lobe volumes and episodic memory. Volumetric data from the dorsolateral (DLPFC), and visual cortices (VC) were obtained from 61 younger and older adults (♀54%). The major homozygote G, T, or A genotypes in single nucleotide polymorphisms (SNPs: rs879606; rs907094; rs3764352, the two latter in complete linkage disequilibrium), at the DARPP-32 regulating *PPP1R1B* gene, influenced frontal gray matter volume and episodic memory (EM). Homozygous carriers of allelic variants with lower DARPP-32 expression had an overall larger prefrontal volume in addition to greater EM recall accuracy after accounting for the influence of age. The SNPs did not influence VC volume. The genetic effects on DLPFC were greater in young adults and selective to this group for EM. Our findings suggest that genomic variation maps onto individual differences in frontal brain volumes and cognitive functions. Larger DLPFC volumes were also related to better EM performance, suggesting that gene-related differences in frontal gray matter may contribute to individual differences in EM. These results need further replication from experimental and longitudinal reports to determine directions of causality.

## Introduction

Both glutamate and dopamine (DA) can influence individual differences in both the recall of memories and the functions of the frontal lobes (O'Carroll and Morris, 2004) that are rich of glutamatergic and dopaminergic cells (Tseng and O'Donnell, 2004). Thirty-two Kilodaltons dopamine- and cAMP-regulated neuronal phosphoprotein (DARPP-32, encoded by *PPP1R1B*) is localized to neurons containing DA receptors and is a mediator of dopamine signaling in part through regulation of protein kinase A (PKA) and protein phosphatase 1 (PP-1). PKA and PP-1 play central roles in the integration between glutamate and dopamine signaling, as well as in regulating activity of other effector molecules (e.g., neurotransmitters; Svenningsson et al., 2004; Gould and Manji, 2005; Fernandez et al., 2006). DARPP-32 influences several signaling pathways in a bidirectional way dependent on its phosphorylation state. DARPP-32 phosphorylated on Thr34 by cAMP-dependent PKA inhibits PP-1, likely by docking into its active site. Conversely, when DARPP-32 is phosphorylated on Thr75 it inhibits PKA hence allowing PP-1 activity. PP-1 activation can also be attained by other protein phosphatases (e.g., PP-2B) that dephosphorylate Thr34. Activation of stimulatory D1 receptors facilitates signaling via the PKA/Thr34-DARPP-32/PP-1 cascade, whereas activation of inhibitory D2 receptors leads to Thr75 phosphorylation and inhibition of PP-1. Glutamate signaling integrates with dopaminergic transmission on DARPP-32 by NMDA and AMPA receptor-driven dephosphorylation of both Thr34 and Thr75 through PP-2B (Nishi et al., 1997; Greengard et al., 1999; Svenningsson et al., 2004; Gould and Manji, 2005; Fernandez et al., 2006).

DARPP-32 activity is also regulated at the genetic level. Genetic variation in *PPP1R1B*, particularly in the minor alleles of the single nucleotide polymorphisms (SNPs) rs879606, rs907094, and rs3764352, have been associated with reduced expression of full-length DARPP-32 mRNA in prefrontal cortex (Meyer-Lindenberg et al., 2007) as well as increased expression level of a transcript encoding a truncated DARPP-32 (tDARPP-32) in the dorsolateral region of the prefrontal cortex (DLPFC; Kunii et al., 2014). The functional effect of these genetic variants may depend on the expression effects of both full-length DARPP-32 and tDARPP-32. In particular, tDARPP-32, containing neither the Thr34 phosphorylation site nor the PP-1 inhibitory domain, which are crucial for brain dopamine signaling, appears to interfere with full-length DARPP-32 inhibition of PKA in a dominant-negative way (Gu et al., 2009). Importantly, DARPP-32 and tDARPP-32 also increase with postnatal age (Kunii et al., 2014), suggesting potential differences across the lifespan. Recent data show that DARPP-32 is released in response to D1/D5 dopamine receptor activation in learning (Karunakaran et al., 2016), which makes genotypes regulating its expression important candidates for episodic memory (EM) functions. Recall of episodic information depends on prefrontal function according to a number of positron emission tomography (PET) and functional magnetic resonance imaging (fMRI) studies (Tulving et al., 1994; Fletcher et al., 1998; Nyberg, 1998). Longitudinal findings further underscores the intimate relationship between EM performance and prefrontal gray matter integrity (Persson et al., 2016).

Indeed, recent imaging genetics studies suggest that genotype variation in *PPP1R1B*, encoding for DARPP-32, can affect function of the DLPFC and gray matter integrity (Meyer-Lindenberg et al., 2007; Curčić-Blake et al., 2012). Those homozygous for the major G or T alleles at SNPs rs879606, rs907094, and rs3764352, with proposed higher full-length DARPP-32, have been reported to have increased intrinsic inferior frontal connectivity in associative learning (Curčić-Blake et al., 2012), increased activation of the DLPFC, and parallel deactivation of the striatum during exposure to tasks tapping higher order cognitive functions (Meyer-Lindenberg et al., 2007) and processing of emotional faces (Curčić-Blake et al., 2012).

The rs879606 A allele has further been associated with EM performance, although this effect did not pass stringent multiple test correction (Houlihan et al., 2009). Other behavioral measures, such as greater trait anger (Reuter et al., 2009) and reward learning (Frank et al., 2007, 2009), have been associated with SNP genotypes coding for DARPP-32. Given that previous observations have been inconclusive regarding the role of the *PPP1R1B* gene, its association with EM and brain function remains to be specified.

### Study aims

We investigated the effects of three SNPs (rs879606, rs907094, and rs3764352) in the gene coding for DARPP-32 on frontal cortex volume and EM function, applying a neurocognitive-genetic approach (Frank and Fossella, 2011) grounded in the outlined genotype effects on DLPFC and higher order cognitive functions (Meyer-Lindenberg et al., 2007; Curčić-Blake et al., 2012). A series of structural equation models with latent variables were performed to assess: (1) direct genetic effects on regional brain volume and EM, and (2) potential differences in the effects as a function of chronological age. The visual cortex (VC) was used as a control region due to its resilience to age-effects on volume and its lack of association with EM performance and DARPP-32 expression. For individuals who are carriers of SNPs that were previously associated with lower DARPP-32 expression, we expected to find larger DLPFC volumes and better memory performance.

## Methods

### Participants

Thirty young (20–31 years, ♀53%) and 31 older adults (65–74 years, ♀54%) were recruited through local media advertisement. All participants were right-handed native Swedish speakers with normal or corrected-to-normal vision. All participants had no history of neurological, psychiatric, or cardiovascular diseases. None of the participants reported any use of psychotropic drugs. Each individual signed an informed consent after the experimental procedures were explained. The study was approved by the Regional Ethical Review Board in Stockholm at the Karolinska institute.

### MRI protocol

#### Imaging acquisitioning

Images were acquired using a 3T Siemens Magnetom Tim Trio scanner (Siemens, Erlangen, Germany) at the Karolinska institute, Stockholm, Sweden, using a 32-channel head coil. To minimize noise while in the scanner, participants were given headphones and earplugs. Head movement was minimized via cushions positioned inside the head coil. For all participants, a radiologist screened the T1- and T2-weighted structural scans to ensure absence of space-occupying lesions and signs of pathology. One hundred and seventy-six slices were acquired in a sagittal orientation. High resolution, T1-weighted MPRAGE anatomical scans were collected using the following parameters: repetition time (TR) = 1,900 ms, echo time (TE) = 2.52 ms, flip angle (FA) = 9 degrees, field of view (FOV) = 256 × 256 mm^2^, voxel size 1 × 1 × 1 mm. FLAIR: TE, 89 ms; TR, 9,000 ms; FA, 130°; inversion time (TI), 2,500 ms; section thickness, 4.0 mm; FOV, 199 × 220 mm.

#### Volumetric measurement

**Figure 1:**
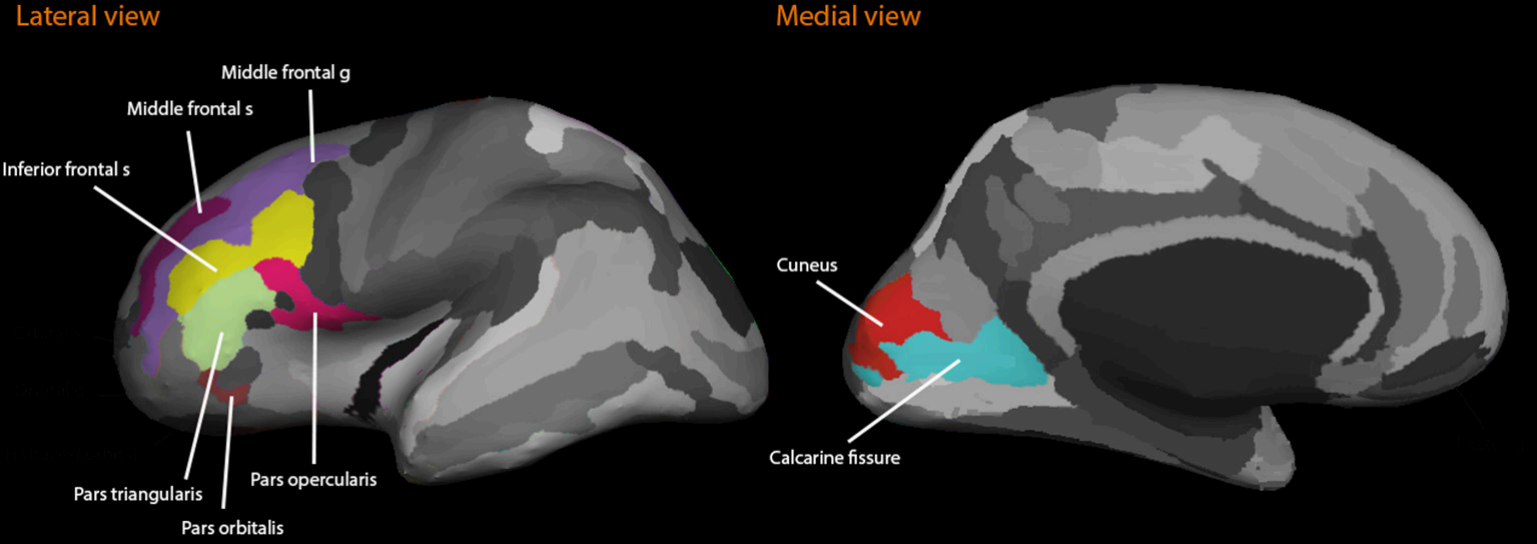
**The figure illustrates the regions of interest included in the dorsolateral prefrontal cortex structure study**. Lateral and medial views are shown.

Freesurfer image analysis suite (version 5.1; http://surfer.nmr.mgh.harvard.edu/) was used for automatic volumetric segmentation, cortical surface reconstruction, and parcellation to quantify the brain volumes of interest (Destrieux et al., 2010). Automated volumetric measures have been validated against histological analysis (Rosas et al., 2002). Using this method, we obtained volumes from the left and right hemispheres from the dorsolateral frontal cortex (the medial frontal sulci and gyri; inferior frontal sulcus; the inferior frontal gyri: pars triangularis, pars opercularis, and pars orbitalis), and the visual cortices (calcarine fissure and cuneus), from the T1-weighted images (see Figure 1). Prior to statistical analyses, all regional volumes were adjusted for the volume of the intracranial vault (ICV) through analysis of covariance (Jack et al., 1989).

### Genotyping

Genomic DNA was extracted from peripheral blood samples and genotyped at the Mutation Analysis Facility at the Karolinska Institute, Huddinge, Sweden in a masked study design. Genotyping was conducted with a single-nucleotide extension reaction, with allele detection by mass spectrometry (SequenomMassArray system; Sequenom, San Diego, CA). Polymerase chain reaction (PCR) and extension primers were designed using the MassArray assay design software. The genotyping success rate for the SNPs rs879606, rs907094, and rs3764352 was 100%. Since rs907094 and rs3764352 were in complete linkage disequilibrium, only rs907094 was considered in the study analyses.

### Cognitive measures

#### Episodic recall

Episodic memory (EM) was measured using a word list that consisted of 16 unrelated nouns. Each of the words was presented to participants both visually and orally, at a presentation rate of 5 s. The participants were instructed to remember the words for a subsequent memory test. Immediately after presentation of the word list, participants were given 2 min to freely recall the words. The outcome variable consisted of the number of correctly recalled words.

## Statistical analyses

A series of structural equation models were carried out to investigate potential effects of rs879606, and rs907094 on episodic recall and prefrontal brain volumes. Homozygosity for the major alleles G or T alleles was coded 1 [e.g., rs879606 G/G (G/G vs. any A: *n* 39/22); rs907094 T/T (TT vs. any C: *n* 33/28)], and heterozygotes/minor homozygotes were coded 0 (e.g., rs879606 AG/AA; CC/TC for rs907094). The coding scheme for the allelic variants was supported by recent findings from histology, reporting dose-specific DARPP-32 expression per allele in the DLPFC (Kunii et al., 2014). Dose effects for each of the three genotypes could not be investigated due to the scarcity of minor allele homozygotes (e.g., rs879606: *n* = 3). Older adults were coded as 1 and younger adults as 0.

**Figure 2:**
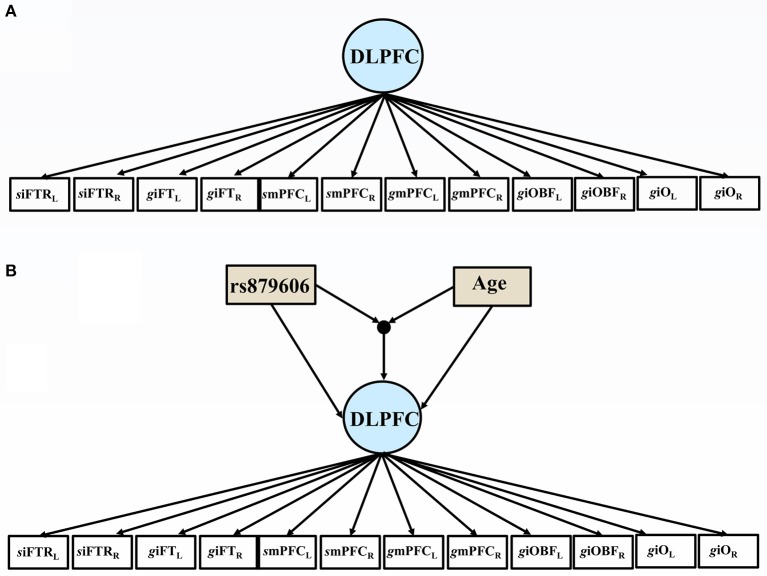
**(A)** The path diagram illustrates the factor model, comprising the bilateral volumes of the dorsolateral prefrontal cortex (DLPFC). Subscript L indicate left hemisphere, and R = right hemisphere; siFTRL = left inferior frontal sulcus; giFTL = left inferior pars triangularis; smPFCL = left medial frontal sulcus; gmPFCL = left medial frontal gyrus; giOBFL = left inferior pars orbitalis; left pars opercularis giOL. **(B)** The path diagram illustrates the effects of the covariates of the volumes of the dorsolateral prefrontal cortex (DLPFC). Subscript L indicate left hemisphere, and R = right hemisphere; siFTRL = left inferior frontal sulcus; giFTL = left inferior pars triangularis; smPFCL= left medial frontal sulcus; gmPFCL = left medial frontal gyrus; giOBFL = left inferior pars orbitalis; left pars opercularis giOL. rs879606 GG were coded 1, and any A allele was coded 0.

First, simple correlations and descriptive statistics were calculated. Second, bilateral volumetric data of gyri and sulci from the left and right hemispheres for each region of interest (ROI) were specified as factors using a confirmatory factor model (CFA) (see Figure 2) in the dorsolateral prefrontal cortex (DLPFC) and the visual cortex (VC) (see Section Volumetric Measurement and Figure 1). Third, age and the SNPs, and the interaction term thereof were added as covariates to predict variations in regional brain volume and episodic memory scores. Following, we stratified the analyses on each age group.

Conventional cut-off criteria for joint evaluation of model fit was considered in evaluation of the models fit to the data: Comparative Fit Index (CFI) >0.95, the Standardized Root Mean Square Residual (SRMR) <0.08, and Root-Mean-Square Error of Approximation (RMSEA) <0.08 (Browne and Cudeck, 1993; Hu and Bentler, 1998), in addition to the χ^2^ test with its degrees of freedom (*df*). We corrected for the False Discovery Rate (FDR) using the Benjamini and Hochberg method (Benjamini and Hochberg, 1995), with the critical level denoted α′ and the nominal significance level of α = 0.05.

### Results

#### Correlations and descriptive statistics

Descriptive statistics are presented in Table 1 and Supplementary Tables 1, 2. The genotype distribution of the single nucleotide polymorphism (SNP) rs879606 (χ^2^ = 0.143, *p* = 0.704), and rs907094 (χ^2^ = 0.01, *p* = 0.931), did not deviate from Hardy–Weinberg equilibrium (HWE). Participants with different allelic variants of the rs879606 and rs907094 did not differ by means of age, sex, or level of education (all *p*'s > 0.05). Zero-order correlations of all variables are presented in Table 2. A positive manifold of correlations was observed over all ROIs. Older age was associated with smaller regional brain volume in all ROIs. Episodic memory (EM) scores showed a positive association with volumes in all ROIs, and effect sizes were strong (≥0.50; Cohen, 1992) for the DLPFC, and moderate for the VC (≥0.30; Cohen, 1992). As seen in Table 3 DLPFC volumes were larger in rs879606 GG carriers compared to A-carriers, while ROI volume was moderately but not statistically significantly related to rs907094 (*p* 0.08) alleles. No effects of any allele were found for VC volume (all *p*'s > 0.05). GG carriers of the rs879606 allele had better EM scores compared to A-carriers, while the behavior—brain volume correlations with the other SNPs genotypes were non-significant (*p* > 0.05).

**Table 1:** **Descriptive statistics**.

	**Min**.	**Max**.	**Mean**	**SD**
**AGE (50.8% OLD)**				
Age (in years)	20	74	46.016	21.906
**SEX (54% WOMEN)**				
Education (years)	9.00	27.00	14.678	3.013
HADS-D	0	6	2	1.789
MMSE	27	30	29.09	.859
DLPFC_cm3_	37.008	61.001	47.002	6.333
VC_cm3_	8.172	16.339	11.542	1.743
EM	4	15	8.573	2.539
rs879606GG (63.9%) rs907094 AA^*^				

Older adults: 65–74 years; (younger adults: 20–30 years); Min, Minimum; Max, Maximum; SD, Standard deviation; HADS-D, Hospital anxiety and depression scale; MMSE, Mini mental state examination; DLPFC, dorsolateral prefrontal cortex; VC, Visual Cortex; EM, Episodic Memory;

* *In complete linkage disequilibrium with rs3764352*.

**Table 2:** **Zero-order correlations among regional brain volumes, memory scores, genetics, and age**.

	**DLPFC**	**VC**	**EM**	**Age**	**rs879606**	rs907094^*^
DLPFC	**0.736**					
VC	**0.547**	1				
EM	**0.496**	**0.386**	1			
Age	−**0.774**	−**0.578**	−**0.570**			
rs879606	**0.262**	0.191	**0.290**	−0.193	1	
rs907094^*^	0.254^**^	0.142	0.100	0.015	**0.815**	**1**

Age: Old = 1, young = 0; Older adults: 65–74 years; (younger adults: 20–30 years); DLPFC, Dorsolateral prefrontal cortex volume; VC, Visual cortex volume; EM, Episodic memory; Age (0 = younger, 1 = older); rs879606 (1 = GG, 0 = AG, AA); rs907094 (1 = TT, 0 = CT, CC);

* *In complete linkage disequilibrium with rs3764352; Significant (p < 0.05) correlations are in bold face*.

** *p = 0.081–0.110*.

**Table 3:** **The effects of age and DARPP-32 coding genotypes on regional brain volumes and episodic memory**.

	**DLPFC**	**VC**	**EM**
***rs879606***			
Age	−0.619 (0.121)^***^	−0.520 (0.183)^**^	−0.291 (0.173)
SNP	0.368 (0.117)^**^	0.052 (0.159)	0.334 (0.155)^**^
SNP × Age	−0.267 (0.143)	−0.033 (0.206)	−0.352 (0.193)
***rs907094ł***			
Age	−0.738 (0.098)^***^	−0.398 (0.155)^**^	−0.440 (0.146)^**^
SNP	0.302 (0.110)^**^	0.201 (0.143)	0.198 (0.147)
SNP × Age	−0.195 (0.136)	−0.218 (0.186)	−0.218 (0.186)

Age (0 = younger, 1 = older); Old = 1, young = 0; Older adults: 65–74 years; (younger adults: 20–30 years); DLPFC, dorsolateral prefrontal cortex; VC, Visual Cortex; EM, Episodic Memory; Age (0 = younger, 1 = older); ł = in total linkage disequilibrium with rs3764352; rs879606 (1 = GG, 0 = AG, AA); rs907094 (1 = TT, 0 = CT, CC); Probabilities (p) are adjusted for false discovery rate using Benjamini–Hochberg correction (α′), with a nominal α = 0.05;

*** *p ≤ 0.01*,

** *p 0.01–0.29; α′ = 0.038*.

#### Confirmatory factor models for the regions of interest

As mentioned previously, two separate CFA models were specified using bilateral volumes from the DLPFC and VC regions; see Figure 2A for an illustration. The models showed a good fit to the data by means of joint criteria of model fit: CFI >0.95, SRMR <0.08, and RMSEA <0.08 (Browne and Cudeck, 1993; Hu and Bentler, 1999). All chi square tests of model fit were non-significant. See Supplementary Table 3 for full information about model fit indices.

#### The effect of covariates

**Figure 3:**
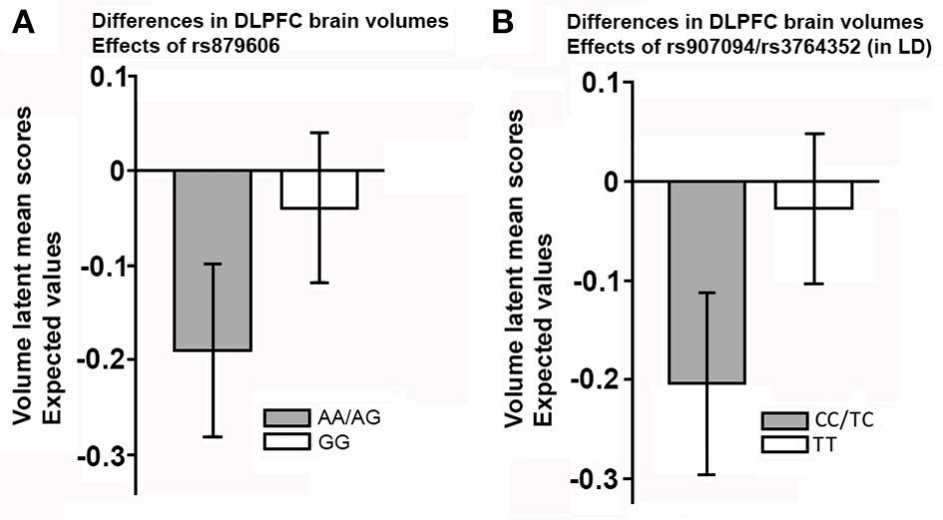
**(A,B)** The effect of the single nucleotide polymorphisms (rs879606, rs907094) on the volumes of the dorsolateral frontal cortex (DLPFC). **(A)** rs879606 **(B)** rs907094. rs907094 is in in complete linkage disequilibrium with rs3764352 and are therefore combined. Homozygosity for major alleles GG-TT marked in white in **(A,B)**, previously associated with higher DARPP-32 expression, relative to heterozygote and homozygote minor allele genotypes (Kunii et al., 2014), and herein associated with larger DLPFC volumes. The scores are factor scores computed from the estimates of the models while taking into account the effects of covariates. The error bars represent 95% confidence intervals of the means.

**Figure 4:**
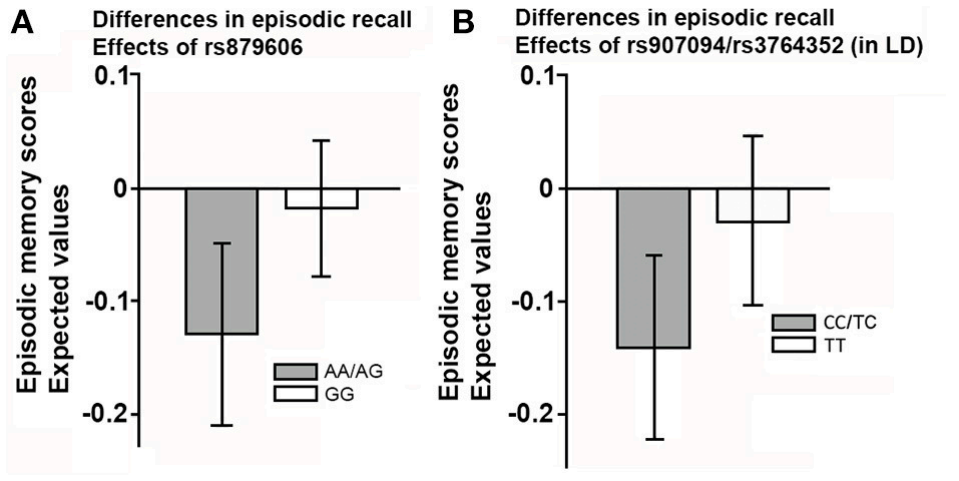
**(A,B)** The effect of the single nucleotide polymorphisms (rs879606; rs907094) on episodic recall in **(A)** rs879606, and **(B)** rs907094 genotypes. rs907094 and rs3764352 are in complete linkage disequilibrium (LD) and are therefore combined. Homozygosity for major alleles GG-TT marked in white in **(A,B)** are associated with higher DARPP-32 expression relative to heterozygote and homozygote minor allele genotypes (Kunii et al., 2014). The scores are factor scores computed from the estimates of the models while taking into account the effects of covariates. The error bars represent 95% confidence intervals of the means.

Since sex and education were both were unrelated to the SNPs studied (all *p*'s > 0.05), we excluded these covariates from further analyses for the sake of model parsimony. See Figure 2B for a visual illustration of the model. All results are presented in Table 3. Older age predicted smaller volumes across all ROIs (all *p'*s 0.0001). Mean differences emerged between younger and older individuals in EM scores, according to the model including rs879606 (*p* = 0.003, α′ = 0.038, Table 3, row 3 column 4), while a trend in the same negative direction was present for the rs907094 model (*p* = 0.092, Table 3, row 3 column 6). Homozygote rs879606 GG-carriers had a larger DLPFC (*p* = 0.002, α′ = 0.038, Figure 3A) volumes compared to homozygotes of the minor allele and heterozygotes (AA/AG). A similar positive relationship was found for homozygous rs907094 TT carriers (*p*'s 0.006, α′ = 0.038, Figure 3B) compared to CC/CT carriers. VC volume was not related to any of the SNP genotypes (all *p*'s > 0.05). The rs879606 GG allele was related to better EM-performance (*p* = 0.029, α′ = 0.038), but the corresponding effect for rs907094 TT genotypes did not reach significance (*p* = 0.110; see Figures 4A,B).

The rs879606 SNP explained 14.3% of the variance in DLPFC volume. The SNPs rs907094 accounted for 4.1% of the variance in DLPFC. The SNPs further explained 5% of the variance in EM performance. None of the age-interactions were significant, although some relationships were at trend level (e.g., *p* = 0.064 for DLPFC, and *p* = 0.068 for EM: α′ = 0.038). Rather, the genotype effects, showing an advantage for larger DLPFC volumes in GG and TT carriers, were present in both younger and older adults when the analyses were further stratified on younger and older adults (*p*'s = 0.001–0.018). Only younger adults showed a positive relation between rs879606 GG genotype and episodic memory recall (rs879606: β = 0.336, SE = 0.162, *p* = 0.038, α′ = 0.038), while the effect was not significant for rs907094 (*p* > 0.05). The relationship between genotype and EM performance was not significant in the older group (*p* > 0.05).

## Discussion

In the present study, the effects of the *PPP1R1B* gene coding for DARPP-32 on dorsolateral prefrontal cortex (DLPFC) volume and episodic memory (EM) were investigated. We were able to replicate the previously reported finding that major alleles of the single nucleotide polymorphisms (SNPs) rs879606 and rs907094 in *PPP1R1B* associate with cognitive function. Homozygous carriers of the rs879606 major G allele performed better on a free recall test of EM compared to A-carriers. This result corroborates previous findings. We, for the first time, shed light on *PPP1R1B*-related variation in DLPFC regional brain volumes. Gene-related differences in frontal gray matter may contribute to individual differences in EM. This is particularly interesting given the role of DLPFC in recall of episodic events and of recent work showing that these SNPs have a genotype dose-specific effect on expression of DARPP-32 in this brain region (Meyer-Lindenberg et al., 2007; Kunii et al., 2014). Particularly, we show that carriers of genotypes associated with higher frontal full-length DARPP-32 expression and lower expression of truncated DARPP-32 had larger DLPFC volumes. Larger DLPFC volumes were also related to higher EM performance, suggesting that DNA-sequence-related expression differences of DARPP-32 in frontal gray matter may contribute to individual differences in EM.

### PPP1R1B association with memory scores

Homozygous carriers of the rs879606 G allele performed better on a free recall test of EM compared to A-carriers. This result corroborates previous findings of a relationship between this particular allele and EM in a large population cohort (Reuter et al., 2009). A similar but much weaker and non-significant relationship to EM was found between for the rs907094 genotypes. The SNPs were previously reported to have similar dose-dependent influence on DARPP-32 expression (Kunii et al., 2014), which should influence the dopamine (DA) system, and thus similarly contribute to EM functioning (Lewis, 2012). The difference in effect size between SNPs in our study might be due to different LD patterns in the study groups, but replication is still warranted.

In addition, the age-stratified analyses revealed that the reported effect was primarily present in the younger sample. The specific reason for this finding is unknown, but the majority of previous reports on DARPP-32 genotype variation in cognitive performance are based on studies on younger adults (Meyer-Lindenberg et al., 2007; Frank et al., 2009; Curčić-Blake et al., 2012), and the maturation of the DA system in younger adults may interplay with the genetic effects.

### PPP1R1B association with brain volumes

We report herein that homozygotes of the major alleles (or haplotype) G-T, vs. other genotypes (or haplotypes), have larger DLPFC brain volumes. This finding is well in line with previous reports showing that greater prefrontal functional network connectivity was associated with these genotypes (Meyer-Lindenberg et al., 2007; Curčić-Blake et al., 2012). Our results also suggest increased EM-related plasticity in DLPFC for persons with genotypes that are associated with higher DLPFC full-length DARPP-32 expression (Meyer-Lindenberg et al., 2007; Kunii et al., 2014). Clearly, DARPP-32 is a hub in several signaling pathways that influence biochemical, electrophysiological, transcriptional, and behavioral effects related to dopaminergic and glutamatergic input to dopaminergic neurons. Thus, larger DLPFC volumes were related to major alleles (GG, TT) as well as better EM performance.

### Limitations

The present results should be interpreted in the context of some limitations. First, the study suffers from limited generalizability due to the non-random recruitment procedure, specifically relying on a sample of convenience. Second, despite the applied exclusion criteria, sub-clinical influence of dementia, or affective, or psychotic disorder may be an issue due to potential pre-clinical disease stages at sub-clinical levels. Moreover, we used a candidate gene approach. Many genetic variants are likely to contribute to heritability of EM and structural integrity of the frontal lobes, but it is unclear which multiple genes are of importance (Stein et al., 2010). Genome wide association studies suggest that SNPs influencing the immune-system may sway EM (Debette et al., 2015). Our sample was too small, however, for a thorough investigation of simultaneous effects of additional genes and interactions among them. However, we had sufficient power to detect small genetic effects on EM, with 5% of variance explained (Cohen, 1992), and small to moderate effect-sizes for frontal brain volumes (≥10%; Cohen, 1992). Potential effects of age magnification needs to be addressed by future reports relaying on larger population based data, as the relatively small sample size may discourage additional influence of older age on the relationship between genotype and neurocognitive function. We did not include direct measures of DARPP-32 level or activity, nor dopamine efficiency, and the specific biochemical mediators of the genetic effects needs to be elucidated in future research by a multimodal imaging protocol that incorporate magnetic resonance spectroscopy or PET to trace such indices. Lastly, a longitudinal design would more accurately address the direction of causality. Cross-sectional inferences do not always apply to longitudinal work due to confounds with between-person age trends, in the former (Lindenberger and Pötter, 1998; Hofer and Sliwinski, 2001). Therefore, our results would benefit from future replications using large-scale longitudinal studies to increase the generalizability of the results.

### Conclusion

We report the importance of *PPP1R1B* genetic variants (rs879606, rs907094, rs3764352), associated with DARPP-32 levels, on EM performance and volume of the dorsolateral region of the frontal lobes.

## Author contributions

NP: Research questions, study design, statistical analyses and interpretation of data, drafting the manuscript, editing and revising the manuscript; HF: Editing, critical revision; JP: Editing, critical revision, and cortical parcellation; CL Editing critical revision.

### Conflict of interest statement

The authors declare that the research was conducted in the absence of any commercial or financial relationships that could be construed as a potential conflict of interest.
