# Supplementary material for: Effects of DARPP-32 Genetic Variation on Prefrontal Cortex Volume and Episodic Memory Performance
Source: Front Neurosci. 2017 May 11;11:244. doi: 10.3389/fnins.2017.00244 (PMC5425487; doi:10.3389/fnins.2017.00244)
Supplement: Supplementary file 1 [file Table1.docx]

|  | GG (*n* = 39) | | | | | GA/AA (*n* = 22) | | | | |
| --- | --- | --- | --- | --- | --- | --- | --- | --- | --- | --- |
|  | Min. | Max. | Mean | SD | % | Min. | Max. | Mean | SD | % |
| Older adults |  |  |  |  | 44 |  |  |  |  | 63 |
| Age (in years) | 20 | 74 | 43.871 | 21.822 |  | 20 | 74 | 52.590 | 21.413 |  |
| Women |  |  |  |  | 44 |  |  |  |  | 63 |
| Education (years) | 9 | 19 | 14.513 | 2.467 |  | 10 | 27 | 14.954 | 3.810 |  |
| HADS-D | 0 | 6 | 2.15 | 1.760 |  | 0 | 6 | 1.77 | 1.850 |  |
| MMSE | 27 | 30 | 29.06 |  |  | 27 | 30 | 29.14 | .834 |  |
| DLPFC | 37.474 | 61.002 | 48.582 | 6.739 |  | 37.008 | 51.568 | 44.273 | 4.512 |  |
| VC | 8.250 | 16.339 | 11.794 | 1.776 |  | 8.172 | 13.798 | 11.107 | 1.632 |  |
| EM | 4 | 15 | 9 | 2.564 |  | 4 | 14 | 7.818 | 2.363 |  |

Supplementary Table 1. Descriptive statistics for the allelic variants in the rs879606 polymorphism.

*Note.* Older adults: 65-74 years; (younger adults: 20-30 years); Min = Minimum; Max = Maximum; HADS-D = Hospital anxiety and depression scale: Depression; MMSE = Mini mental state examination; DLPFC= dorsolateral prefrontal cortex; VC= Visual Cortex; EM = Episodic Memory.
