# Supplementary material for: Effects of DARPP-32 Genetic Variation on Prefrontal Cortex Volume and Episodic Memory Performance
Source: Front Neurosci. 2017 May 11;11:244. doi: 10.3389/fnins.2017.00244 (PMC5425487; doi:10.3389/fnins.2017.00244)
Supplement: Supplementary file 2 [file Table2.docx]

|  | DLPFC | VC | EM | Age | rs879606 | rs907094^*^ |
| --- | --- | --- | --- | --- | --- | --- |
| DLPFC | **.736** |  |  |  |  |  |
| VC | **.547** | 1 |  |  |  |  |
| EM | **.496** | **.386** | 1 |  |  |  |
| Age | **-.774** | **-.578** | **-.570** |  |  |  |
| rs879606 | **.262** | .191 | **.290** | -.193 | 1 |  |
| rs907094^*^ | .254** | .142 | .100 | .015 | **.815** | **1** |

Ta­ble 2. Zero-order correlations among regional brain volumes, memory scores, genetics and age.

Note. Age: Old =1, young =0; Older adults: 65-74 years; (younger adults: 20-30 years); DLPFC = Dorsolateral prefrontal cortex volume; VC = Visual cortex volume; EM = Episodic memory; Age (0 = younger, 1 = older); rs879606 (1 = GG, 0 = AG, AA); rs907094 (1 = TT, 0 = CT, CC); ^*^in complete linkage disequilibrium with rs3764352; Sig­nificant (*p* < .05) correlations are in bold face. ** *p* = .081-.110.
