# Supplementary material for: Effects of DARPP-32 Genetic Variation on Prefrontal Cortex Volume and Episodic Memory Performance
Source: Front Neurosci. 2017 May 11;11:244. doi: 10.3389/fnins.2017.00244 (PMC5425487; doi:10.3389/fnins.2017.00244)
Supplement: Supplementary file 3 [file Table3.docx]

|  | RMSEA | CFI | SRMR | Chi-Square (*df*) |
| --- | --- | --- | --- | --- |
| DLPFC | .060 (.000-.010) | .960 | .067 | 63.3153(52), *p* = .364 |
| VC | .000 (.000-.115) | 1.000 | .012 | .224 (2), *p* = .894 |

Supplementary Table 3. Model fit indices for the measurement models.

*Note.* RMSEA = Root Mean Square Error of Approximation, 90% confidence interval are presented in parentheses; CFI = Bentler comparative fit index; SRMR = Standardized Root Mean Square Residual, 90% confidence interval are presented in parentheses; *df* = degrees of freedom; DLPFC= dorsolateral prefrontal cortex; VC= Visual Cortex.
